# Hypothesizing Nutrigenomic-Based Precision Anti-Obesity Treatment and Prophylaxis: Should We Be Targeting Sarcopenia Induced Brain Dysfunction?

**DOI:** 10.3390/ijerph18189774

**Published:** 2021-09-16

**Authors:** Kenneth Blum, Mark S. Gold, Luis Llanos-Gomez, Rehan Jalali, Panayotis K. Thanos, Abdalla Bowirrat, William B. Downs, Debasis Bagchi, Eric R. Braverman, David Baron, Alphonso Kenison Roy, Rajendra D. Badgaiyan

**Affiliations:** 1 Center for Psychiatry, Medicine & Primary Care (Office of the Provost), Division of Addiction Research & Education, Western University Health Science, Pomona, CA 91766, USA; dbaron@westernu.edu; 2 Institute of Psychology, ELTE Eötvös Loránd University, Kazinczy u. 23-27, 1075 Budapest, Hungary; 3 Division of Nutrigenomics, Genomic Testing Center Geneus Health, San Antonio, TX 78249, USA; 4 Department of Psychiatry, University of Vermont, Burlington, VT 05401, USA; 5 Department of Psychiatry, Wright University Boonshoff School of Medicine, Dayton, OH 45377, USA; 6 Division of Precision Nutrition, Victory Nutrition International, Bonita Springs, FL 34135, USA; billd@vni.life (W.B.D.); debasis@vni.life (D.B.); 7 The Kenneth Blum Behavioral & Neurogenetic Institute, Division of Ivitalize Inc., Austin, TX 78701, USA; luisllanos522@gmail.com (L.L.-G.); Rjalali@ivitalize.com (R.J.); 8 Division of Clinical Neurology, Path Foundation NY, New York, NY 10010, USA; pathmedical@gmail.com; 9 Department of Psychiatry, School of Medicine, Tulane University, New Orleans, LA 70118, USA; drmarksgold@gmail.com (M.S.G.); aroy6@tulane.edu (A.K.R.III); 10 Department of Psychology & Behavioral Neuropharmacology and Neuroimaging Laboratory on Addictions (BNNLA), Research Institute on Addictions, University at Buffalo, Buffalo, NY 14260, USA; thanos@buffalo.edu; 11 Department of Molecular Biology, Adelson School of Medicine, Ariel University, Ariel 40700, Israel; bowirrat@gmail.com; 12 Department of Pharmaceutical Sciences, College of Pharmacy & Health Sciences, Texas Southern University, Houston, TX 77004, USA; 13 Department of Psychiatry, South Texas Veteran Health Care System, Audie L. Murphy Memorial VA Hospital, Long School of Medicine, University of Texas Medical Center, San Antonio, TX 78249, USA; badgaiyan@gmail.com

**Keywords:** obesity, nutrigenomics, resting-state functional connectivity, sarcopenia, BMI, percent body fat, hypodopaminergia, Reward Deficiency Syndrome (RDS)

## Abstract

**Background:** The United States Centers for Disease Control and Prevention (CDC) estimates a total obesity rate of 30% for 12 states and a 20% obesity rate nationwide. The obesity epidemic continues to increase in spite of preventative measures undertaken worldwide. Pharmacological treatments promise to reduce total fat mass. However, medications may have significant side effects and can be potentially fatal. **Data Retrieval**: This brief review, based on a PUBMED search of the key terms “Obesity” and” Sarcopenia,” will present evidence to corroborate the existence of Reward Deficiency Syndrome (RDS) in obesity and the involvement of catecholaminergic pathways in substance seeking behavior, particularly as it relates to carbohydrates cravings. **Expert Opinion:** The genetic basis and future genetic testing of children for risk of aberrant generalized craving behavior are considered a prevention method. Here we present evidence supporting the use of precursor amino acid therapy and modulation of enkephalinase, ***MOA***, and ***COMT*** inhibition in key brain regions. Such treatments manifest in improved levels of dopamine/norepinephrine, GABA, serotonin, and enkephalins. We also present evidence substantiating insulin sensitivity enhancement via Chromium salts, which affect dopamine neuronal synthesis regulation. We believe our unique combination of natural ingredients will influence many pathways leading to the promotion of well-being and normal healthy metabolic functioning. Sarcopenia has been shown to reduce angiogenesis and possible cerebral blood flow. Exercise seems to provide a significant benefit to overcome this obesity-promoting loss of muscle density. **Conclusion:** Utilization of proposed nutrigenomic formulae based on coupling genetic obesity risk testing promotes generalized anti-craving of carbohydrates and can inhibit carbohydrate bingeing, inducing significant healthy fat loss and relapse prevention.

## 1. Introduction

“Weight gain,” “weight management,” and “weight loss” are the most typically used terms referring to fluctuations in body composition, particularly as they relate to total fat mass. Our review, conducted using PUBMED, will provide evidence demonstrating that the focus of “weight” as a meaningful criterion is misguided and deviates from the natural order of processes that leads to improved body and metabolism recomposition. We believe that focusing on weight as a primary outcome leads to counterproductive tactics that hinder the goal of improving suboptimal metabolic functioning. Furthermore, the relevance of fat as espoused by conventional weight loss methods is also misguided as fat is one of the lightest macromolecules in the body and its reduction is an effect of improved metabolic processes that precede its reduction. Genetic, lifestyle, and environmental factors all converge to produce the metabolic efficiency of an individual at any given moment [1]. Conventional weight-loss tactics have failed to produce long-lasting changes in body weight and have ignored the fundamental prerequisite of improving metabolic function before targeting total fat mass reduction. Commercialized “weight loss” regimens, including those guided by medical personnel, fail to consider the concept of a “bi-phasic” genetically mediated “set point” that governs the mammalian response to starvation threats during the first phase of weight loss.

Furthermore, commonly prescribed tactics over-emphasize caloric intake and ignore caloric density and nutrient quality factors more relevant to metabolic efficiency than net caloric intake alone. Finally, current strategies also fail to consider the addictive tendencies of many individuals attempting to enhance body composition [2]. Thus, this review will provide evidence for introducing a technology that will lay the foundation of a novel approach to target healthy, long-lasting outcomes in body recomposition and result in sustainable body mass management.

We propose utilizing a unique natural formula to improve body recomposition and promote healthy, sustainable body mass goals. Our unique nutraceutical formula couple’s synergistic ingredients that address disfunction in the metabolic energy management system. This nutrigenomic approach to body recomposition enhancement uniquely addresses the brain’s reward circuitry by balancing neurotransmitters in key areas of the brain that result in maladaptive cravings for food, especially sugar and carbohydrates. Furthermore, this patented nutraceutical also targets immune system enhancement, homeostasis in both the stress and inflammation management systems, and the neuroendocrine network. These five systems are interrelated and, when balanced, result in optimized metabolic function.

This nutraceutical technology, the first of its kind, is genetically tailored to a patient’s unique genetic makeup and promotes healthy fat loss and metabolic optimization without inducing the Yo-Yo weight gain effects seen in other weight loss regimens. In addition, the use of this technology juxtaposes conventional weight-loss methods in that weight gain may manifest before fat loss due to an increase in muscle mass resulting from enhanced cellular responses to energy metabolism [3].

Our proposed “weight-loss” strategy may encounter one critical barrier—attrition during the early stages of program implementation due to fat-loss occurring in the last stage of improved metabolic activity. We, therefore, stress the significance of education in elucidating the proper phases of body recomposition. We hold the purview that an accurate sequence of events regarding a healthy metabolism begins with water weight loss, followed by increased weight (resulting from increased muscle mass), and finally visible and healthy fat loss [4].

**Figure 1:**
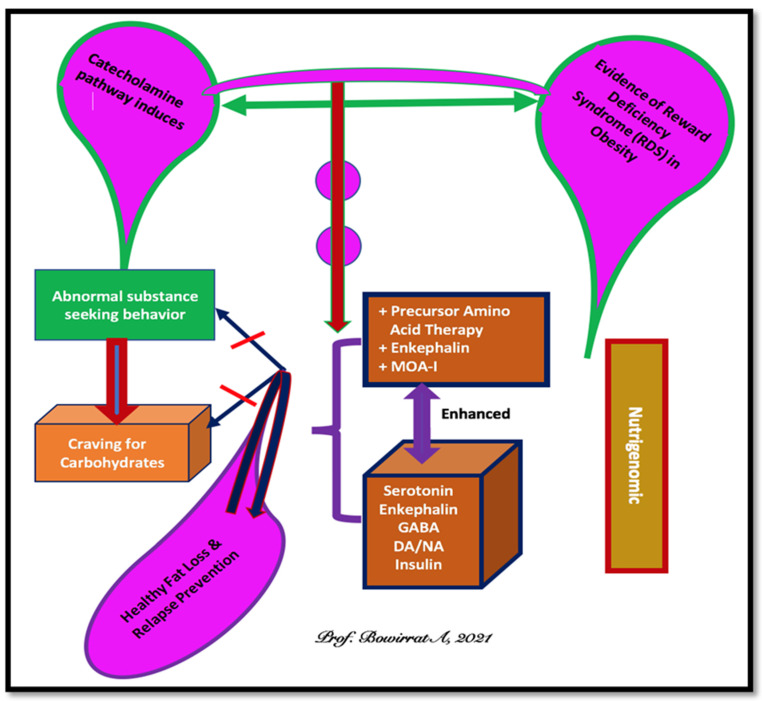
Neuro-Adaptogen and Amino-Acid-Therapy (NAAT) ^TM^ as a putative anti-obesity complex.

Various minerals are implicated in the metabolic processes that promote healthy carbohydrate metabolism, energy production, insulin function, fat oxidation, blood lipid metabolism, and serotonin release, which lead to successful body composition management efforts that result in fat loss. Research into the potential need prompted the development of the present Neuroadaptive-Amino-Acid Therapy formulae for reward dependence. We propose that the best approach to treat and prevent relapse weight gain is through natural, non-toxic nutrigenomic compounds (see Figure 1).

The effectiveness of the nutraceutical technology [5] presented herein provides significant evidence that the term “weight loss” is mischaracterized and, therefore, does not represent an accurate proxy of the desired end goal of attaining healthy long-term metabolic functioning. The term “weight loss” (or any terms using the “weight” language reference) appearing in quotations are deliberately misapplied to emphasize the point that standard tactics and language contribute to inaccurate yet universally accepted dogma regarding effective body recomposition efforts. Current “weight loss” strategies typically overemphasize caloric intake reduction aimed at decreasing total fat mass. The following list includes various weight-loss strategies commonly used today:

- Central Nervous System Stimulants (CNSS) that artificially stimulate the rate of calorie burning (Basal Metabolic Rate [BMR]).
- Appetite Suppressants
- Fat Blockers
- Starch Blockers
- Diuretics (Water Pills)
- Low Calorie Diets
- Low Food Diets
- Meal Replacement Programs (Diet Shakes, bars, etc.)
- High Protein Diets
- High Carbohydrate Diets
- Low/No Carbohydrate Diets
- Low-Fat Diets
- Pre-Meal Fiber/Water “Fill-You-Up” Programs
- Fruit and Fruit juice “Rapid “weight loss”” Programs
- Overnight “weight loss” Programs
- Vegetable Soup Diet Programs
- Liposuction
- Radical Digestive Tract Surgeries
- Acupuncture
- Laxatives
- Hypnosis

Many of these interventions are prescribed individually or combined in efforts to achieve fast “weight loss” outcomes. As previously stated, the primary aim of these interventions revolves around “weight loss” or image enhancement. Such objectives are typically pursued without regard for current information regarding their effects on health and the body’s natural genetically mandated homeostatic response. We know that depriving the body of essential resources is counterproductive as such deprivations simulate the circumstances of famine and lead to genetically programmed energy conservation measures by the body.

Nutrient deprivation induces the body to generate metabolic processes that increase appetite to offset perceived nutrient deficiencies. Strikingly, many of these tactics are approved, supervised, and administered by medical or health professionals. Initially, such tactics appear to promote “weight loss” (phase 1). Eventually, however, these techniques are destined to fail as the metabolism counteracts the perceived nutrient deficiencies with gene-induced adjustments in fat storage and energy management systems. Such recalibrations result in lower basal metabolic rates and increased energy storage via increased fat adipose tissue (phase 2). The inadequacy of such tactics to lose weight are so prevalent that they result in what is now referred to as the “Yo-Yo Weight Gain Effect.” This phenomenon often results in ever-increasing frustration, anxiety, and a sense of helplessness caused by the out-of-control “weight loss” weight gain rebound phenomenon.

A key feature of habits manifesting in obesity is reduced movement and low degrees of physical activity. We know that dopaminergic pathways are critical in the initiation of movement. For example, bradykinesia (slowness of movement) has been attributed to the absence of the murine dopamine D2 receptor gene (***Drd2***) and to hypothermia, implicated in obesity. In addition, a Ser311Cys mutation of ***Drd2*** receptor results in pronounced dopamine receptor disfunction and is implicated with higher BMI scores in specific populations.

Tataranni et al. [6] measured total energy expenditure in 89 non-diabetic Pima Native Americans and reported that homozygotes for the Cys311 allele expended 244 kcal/day less than individuals who were heterozygous and homozygous for the Ser311-encoding allele (*P* = 0.056). Furthermore, 320 non-diabetic Pima Indian homozygotes for the Cys311-encoding allele exhibited 87 kcal/day lower 24 h resting energy expenditure (respiratory chamber) compared to Pima individuals who were homozygous and heterozygous for the Ser311-encoding allele (*P* = 0.026). Consequentially Tataranni et al. were the first to provide evidence that reduced energy expenditure was directly associated with a genetic mutation in humans. In support of this notion, Jenkinson et al. reported higher BMI scores in Heterozygotes at the Ser311Cys ***Drd2*** polymorphism.

## 2. Brain Reward Mechanisms and Nutrigenomic Solutions

Ultimately, obesity results from a lowered basal metabolic rate, increased appetite focused on calorically dense foods (sugars and fats), and upregulation of fat reserves, all of which are hallmark features of nutrient deficiency-induced famine disorder. Thus, attempts to induce weight loss via caloric reduction result in upregulation of genes programmed to resist body fat loss, which ultimately manifests in the previously mentioned “Yo-Yo” phenomenon as indicated in the ***“Thrifty Gene”*** hypothesis [7] (which emphasized the importance of insulin sensitivity). Thus, programmed genetic predispositions triggered by diet-induced weight-loss strategies (those previously mentioned) may lead to the downregulation of resting metabolic rate (RMR). In addition, overconsumption of high-calorie, nutrient-deficient food contributes to weight gain and obesity.

Resistance to the hormone leptin is a feature common to obesity [8,9]. A literature review conducted by Izquierdo et al. concluded that despite 25 years of research revealing that leptin can effectively reduce food intake and body weight, however, a lack of understanding of the mechanisms involved in leptin resistance remains. Leptin is a hormone; by itself, it cannot penetrate the blood–brain barrier, and consequently, it is not utilized for treatment.

Building on previous efforts to understand the nature of obesity, our group, utilizing the first-ever attempt to incorporate DNA-guided therapy to treat and or prevent obesity, has provided evidence to support our hypothesis as presented herein [10]. A total of 1058 subjects were genotyped and administered a KB220Z variant (AKA LG839) contingent upon their polymorphic results. A subset of 27 obese subjects of Dutch ancestry (self-identified) displayed the same DNA schemata of four out of five candidate genes tested (chi-square analysis). Simple t-tests comparing several weight management parameters before and after 80 days of treatment with LG839 were executed. Significant results were witnessed for appetite suppression, sugar craving reduction, snack reduction, reduction of late-night feeding, and weight loss (all *P* < 0.01), increased perception of overeating, improved sleep quality, increased perceived happiness (all *P* < 0.05), and upregulated energy (*P* < 0.001). Multiple polymorphic correlations were identified for genes ***(PPAR-gamma2, LEP, 5-HT2A, MTHFR, and Drd2*** genes) with positive clinical metrics evaluated in this study. Out of the genes tested, only the ***Drd2*** gene polymorphism (A1 allele) displayed a significant Pearson Correlation with the number of days on treatment (r = 0.42, *P* = 0.045). Our group obtained similar outcomes suggesting that DNA-directed targeting of specific modulator genes, in tandem with individualized nutraceutical therapy, offers a unique framework from which to target obesity reduction strategically [11,12,13,14].

Insulin has been demonstrated to increase leptin secretion. Conversely, acute leptin infusion reduces insulin secretion. Park et al. [15] found that acute ICV leptin administration in rodents suppressed the first and second-phase insulin secretion at hyperglycemic clamp by 48% (compared to the control), resulting in reduced insulin resistance. Evidence suggests that insulin resistance is also a primary contributor to obesity. Resistance-induced hyperinsulinemia can provoke leptin-resistant hyperleptinemia with a significant upregulation of white lipid synthesis and storage in adipose tissue, a characteristic of Metabolic Syndrome X. Furthermore, a relationship between the rate of leptin secretion and intracellular ATP concentration appears to exist in adipocytes from obese animals, which results in a positive correlation between leptin and percent body fat. Risk factors for weight gain in obese individuals are low levels of physical activity, low resting metabolic rate (RMR), and reduced lipid oxidation rates [16]. It has been demonstrated that a reduction in body weight as fat mass and fat-free mass is accompanied by a more significant decrease in resting energy expenditure and fat oxidation [17,18,19].

Efficient body recomposition strategies targeting energy modulating systems should simultaneously enhance insulin sensitivity, fat oxidation, serotonin, resting metabolic rate, and reduce appetite (given proper nutritional intake). Our proposed method is designed to target at least five key systems critical for effective weight management by addressing nutrition and is listed in the following section (see Table 1).

Deficiencies in gene expression and nutrition in the reward neurochemical pathway limit the number of necessary neurotransmitters in areas associated with motivated behavior, leading to the manifestation of “Reward Deficiency Syndrome” (RDS). RDS can result in maladaptive cravings for carbohydrates [20]. A dysfunction in the Brain Reward Cascade is the main culprit behind RDS, characterized by abnormal craving behavior to illicit substances, natural substances such as sugar, and behaviors typically appropriated to “impulsive” individuals (excessive shopping, gambling, and risk-taking). Anomalies in the ***Drd2*** and other dopaminergic genes ***(D1, D3, D4, DAT1, COMT, MAOA***) [21] have been implicated in the RDS phenomena. Dopamine, an essential neurotransmitter, is known to moderate feelings of well-being.

**Figure 2:**
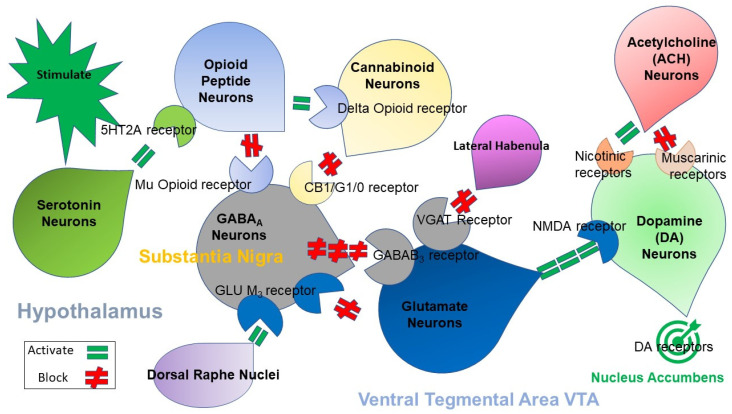
The Brain Reward Cascade.

This sense of well-being is produced via an intricate network of neurological systems that utilize dopamine, serotonin, opioids, and other powerful brain chemicals to instill feelings of satisfaction. We know that depression is associated with low serotonin levels and high levels of endorphins (the brain’s opioids) are implicated with a sense of well-being. The complex system between these critical neurotransmitters regulates dopaminergic activity in the brain reward center and was coined by Blum as “The Brain Reward Cascade” [22] (see Figure 2 [21]).

**Figure 3:**
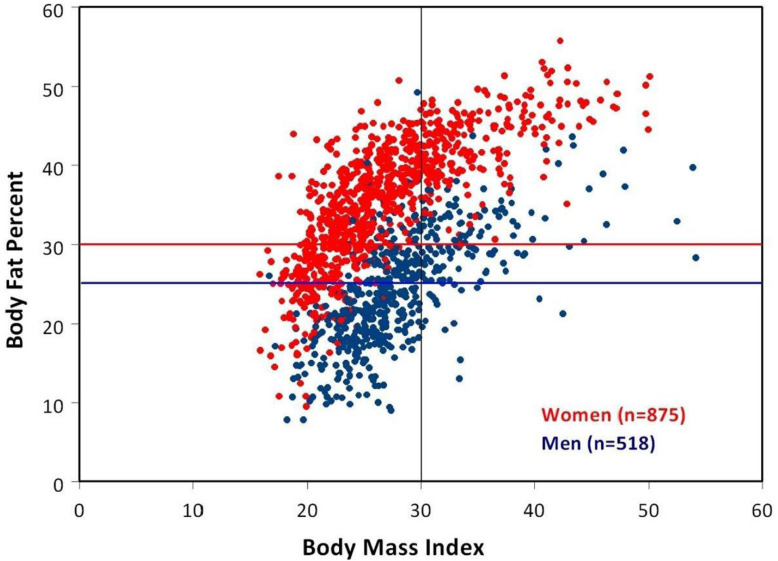
BMI versus percent body fat in the scatter plot.

Figure 3 illustrates the interaction of at least six major neurotransmitter pathways involved in the Brain Reward Cascade (BRC). In the hypothalamus, environmental stimulation causes the release of serotonin, which in turn, via, for example, 5HT-2a receptors, activate (green equal sign) the subsequent release of opioid peptides from opioid peptide neurons, also in the hypothalamus. Then, in turn, the opioid peptides having two distinct effects, possibly via two different opioid receptors: A) inhibits (red hash sign) through the mu opioid receptor (possibly via enkephalin) and projecting to the Substania Nigra to GABAA neurons B) stimulates (green equal sign) Cannabinoid neurons (e.g., Anandamide and 2-archydonoglcerol) through Beta–Endorphin linked delta receptors, which in turn inhibits GABAA neurons at the substania nigra. Cannabinoids primarily 2-archydonoglcerol, when activated, can also indirectly disinhibit (red hash sign) GABAA neurons in the Substania Nigra through activation of G1/0 coupled to CB1 receptors. Similarly, Glutamate neurons located in the Dorsal Raphe Nuclei (DRN) can indirectly disinhibit GABAA neurons in the Substania Nigra through activation of GLU M3 receptors (red hash sign). GABAA neurons, when stimulated, will, in turn, powerfully (red hash signs) inhibit VTA glutaminergic drive via GABAB 3 neurons. It is also possible that stimulation of ACH neurons that at the Nucleus Accumbens ACH can stimulate both muscarinic (red hash) or Nicotinic (green Hash). Finally, Glutamate neurons in the VTA will project to dopamine neurons through NMDA receptors (green equal sign) to preferentially release dopamine at the Nucleus Accumbens (NAc), shown as a bullseye indicating euphoria (a wanting response). The end result is that as dopamine is released (low = unhappiness-endorphin deficiency) and (normal = happiness), depends on the happiness tonic set point [22] [With permission by Blum et al.].

Women (red) who fall above the red line are obese according to the American Society of Bariatric Physicians criteria (DXA percent body fat: ≥30%). Men (blue) who fall above the blue horizontal line are obese according to the American Society of Bariatric Physicians criteria (DXA percent body fat: ≥25%). The upper left quadrant bordered by a red horizontal line (body fat percent = 30%) and black vertical line (BMI = 30) demonstrates the large number of women misclassified as “non-obese” by BMI yet “obese” by percent body fat [with permission from Shar and Braverman].

Anomalies in the ***Drd2*** structure result in decreased dopamine receptor sites in the brain’s reward center. This leads to reduced effectiveness of dopamine communication between critical rewards sites [23,24,25]. Along these lines, Gluskin et al. [24] identified 21 studies examining 19 variants in 11 genes. Specifically, fixed- and random-effects meta-analyses of this variant (5 studies, 194 subjects total) revealed that striatal BP was significantly and robustly lower among carriers of the minor allele (rs1800497, Glu713Lys, also called ‘***Taq1A***’) relative to significant allele homozygotes (*P* = 0.0002) supporting Blum and Noble’s first report [23].

Individuals possessing the Dopamine Receptor Gene variant tend to be serious cocaine abusers [26] and are predisposed to unhealthy appetites that can lead to obesity or overeating [27]. Conversely, these individuals are predisposed to develop anorexia and have high levels of stress over an extended period. Their addiction-prone neuro-circuitry manifests in maladaptive generalized craving behaviors leading to overconsumption of substances such as cocaine, alcohol, nicotine, and glucose (substances known to activate the NAc [addiction center of the brain]) in efforts to activate dopaminergic signaling in reward pathways. Such behaviors serve as a form of self-medication to offset their low D2 receptors caused by genetic antecedents (such as the D2 receptor gene ***Taq1 A1 allele*** [28]). While the scientific community is very concerned about childhood obesity [29], it is also known that there is a concomitant reduction of D2 receptors and potential increased RDS behaviors along with aging.

An investigation by Thanos et al. [29] assessed body weight and locomotor activity in mice throughout their lifespan. They concluded that an enriched environment (EE) was found to be associated with a longer lifespan in mice with regular or decreased expression of the D2 gene. ***Drd2*** +/+ EE mice lived nearly 16% longer than their deprived environment (DE) counterparts. ***Drd2*** +/+ and ***Drd2*** +/− EE mice lived 22% and 21% longer than ***Drd2*** −/− EE mice, respectively. In addition, they found that environmental factors moderated both body weight and locomotor activity. In fact, EE mice show more significant behavioral variability between genotypes than DE mice concerning body weight and locomotion.

Suboptimal dopaminergic activity in the brain reward center results in behaviors that increase the release of dopamine [30]. Such behaviors include the overconsumption of carbohydrates, alcohol, nicotine, and illicit drug use such as cocaine. Low dopamine may also manifest in non-substance-seeking behavior that results in dopamine release such aggression, resulting from abnormalities in specific dopaminergic genes [31]. Carriers of an under-active ***Drd2 Taq1A*** polymorphism who reported childhood sexual abuse were reported to engage in more sensation-seeking behavior than individuals expressing different combinations of alleles who reported no previous abuse exposure. Furthermore, individuals who inherited an under-active allele of ***COMT*** had lower impulsivity scores and displayed lowered frequencies of binge eating behaviors than individuals who lacked the low functioning allele [32]. Thus, RDS can be viewed as a form of biochemically induced sensory deprivation resulting from dopaminergic genetic anomalies [33,34]. The consequences of RDS may manifest on a continuum from mild to severe that interfere with an individual’s capacity to derive pleasure or reward from ordinary day-to-day activities.

## 3. Body Mass Index (BMI) vs. Percent Body Fat

The body mass index (BMI) is commonly utilized to identify adiposity. However, there is controversy suggesting that BMI is an inaccurate obesity classification strategy that underestimates the obesity epidemic and leads to inefficient treatment. Shah and Braverman [35] analyzed the accuracy of specific biomarkers and dual-energy x-ray absorptiometry (DXA) to assist in obesity diagnosis and prophylaxis.

Adiposity in mid-life is significantly associated with reducing life expectancy in women [36]. Moreover, estimates from NHANES, a nationally representative health examination survey, estimated that 34% of adult Americans are overweight (defined as a BMI between 25–30 kg/m^2^), and 34% meet the criteria for obesity (BMI >30 kg/m^2^) [37]. However, the antiquated BMI formula [BMI = weight in pounds/(height in inches)2 × 703], calculated nearly 200 years ago by Quetelet, was not an accurate proxy for metabolic health and was simply an imprecise mathematical estimate [38]. Critically, BMI fails to account for factors affecting adiposity. For example, greater degrees of muscle mass atrophy contributing to sarcopenic obesity in women result increasingly with age. BMI fails to account for this factor and exacerbates inaccurate misclassifications [39]. In addition, men’s BMI scores do not account for the inverse relationships between muscular strength and mortality [40] and fail to adjust to men’s decreased muscle mass loss compared to women as they age.

In the Braverman and Shah [35] cross-sectional analysis of adults with BMI, DXA, fasting insulin, and leptin, results were measured from 1998–2009. The subjects partaking in their study included 37% males, 63% females, 75% white, with a mean age = 51.4 (SD = 14.2). Mean BMI was 27.3 (SD = 5.9), and mean percent body fat was 31.3% (SD = 9.3). As a conclusion to the study, it was reported that BMI characterized 26% of the subjects as obese, while DXA indicated that 64% were obese. A total of 39% of the subjects were classified as non-obese by BMI. Conversely, DXA analysis characterized those same subjects as obese. Furthermore, BMI misclassified 48% of women and 25% of men. Interestingly, a strong significant correlation was detected between increased body fat and increased leptin. The fundamental message is that the investigation conducted by Braverman and Shah [35] demonstrated the prevalence of inaccurate misclassifications and false-negative BMIs in women of advancing age and the reliability of gender-specific revised BMI parameters. In essence, the concept of BMI fails to capture an accurate obesity representation by underestimated its prevalence, particularly in women with elevated leptin levels (>30 ng/mL). Braverman and Shah [35] proposed that clinicians use leptin-revised levels to increase BMI accuracy estimates of percent body fat when DXA is unavailable (see Figure 3 [35]).

## 4. Sarcopenia and Brain Function

Sarcopenia, a term coined by Irwin Rosenberg in 1989 [41,42], was clinically defined as a reduction in strength and skeletal muscle mass resulting from age 43. Sarcopenia has multiple contributing factors: aging, poor diet, lack of sleep, sedentary lifestyle, chronic disease, specific medications, early life development, and early-life environmental factors [43,44,45,46]. Sarcopenia results in an impaired state of health that can lead to a higher incidence of falls, increased risk of fractures, mobility disorders, impaired day-to-day activities, disabilities, loss of independence, and increased risk of fatality [47].

Gustav Fritsch and Edvard Hitzig are responsible for identifying the Motor Cortex as the epicenter of the sequence responsible for muscle contraction. This discovery serves as the basis for the connection between the brain and Sarcopenia [48]. Here, we hypothesize some mechanisms responsible for the deterioration of the relationship between the brain and muscles.

Among the variables that may cause the reduction in neuromuscular signaling is the deterioration of the quality of motor neurons, possibly due to an increase in amyloid-like tangles found in the brain of ordinary aging people. It is known that sporadic cerebral amyloid angiopathy is commonly seen in the elderly who lack detectable symptoms of Alzheimer’s disease [49,50]. This factor cannot be overlooked when examining possible mechanisms that impact how neurons affect the contractile force of muscles. We also know that aging neurons display reduced levels of mitochondrial biogenesis [51] as well as reduced function at neuromuscular junction sites [52]. Another factor affecting the brain to muscle signal transduction is a reduction in the integrity of the myelin sheath [53] (which may be treatable with nutrigenomic interventions containing Lion’s Mane Mushroom) [54]. These factors and likely others may underly mechanisms concerning the relationship between the brain and the onset of Sarcopenia.

One mechanism that potentially contributes to microvascular rarefaction is an age-associated reduction of angiogenesis [55] associated with Sarcopenia. There are several reasons related to microvascular rarefaction in age-related impairment of angiogenesis [56,57,58,59,60,61,62,63,64,65,66,67,68,69,70]. Moreover, Sarcopenia and lower extremity Peripheral Artery Disease (PAD) have musculoskeletal consequences that reduce a patients’ quality of life. Notably, both PAD and Sarcopenia result in elevated oxidative stress, inflammation, skeletal muscle mitochondrial impairments, activation of molecules associated with muscle degradation, and inhibition of specific pathways that modulate muscle synthesis or protection (i.e., IGF-1, RISK, and SAFE), [70]. It is widely accepted that endurance, muscle mass, and strength are required for quality of life and that skeletal muscle microcirculation plays a significant role in muscle health. The link between capillary supply and muscle fiber size is reflected by angiogenesis and hypertrophy and the cross-communication between satellite cells and capillaries. It is known that a dense vascular network is more critical for the rapid repair of muscle damage than the number of satellite cells.

Furthermore, a lower capillary density may also attenuate the hypertrophic response. In addition, capillary rarefaction occurs during aging and during conditions where endothelial apoptosis precedes muscle atrophy. Most importantly, Hendrickse and Degens [71] proposed that capillary rarefaction precedes Sarcopenia. One crucial biological marker related to Sarcopenia, obesity, and aging is the family of Oxylipins. These essential molecules refer to a large class of signaling lipids implicated in the regulation of several biological functions that become dysregulated as a consequence of aging. The Oxylipin signatures of sarcopenic subjects display subtle activation of inflammatory resolution pathways, oxidative stress, inhibition of angiogenesis, and coagulation processes. Heat maps reveal that oxylipins are lost in sarcopenic subjects [72].

## 5. Sarcopenia and Obesity: Can We Treat?

**Figure 4:**
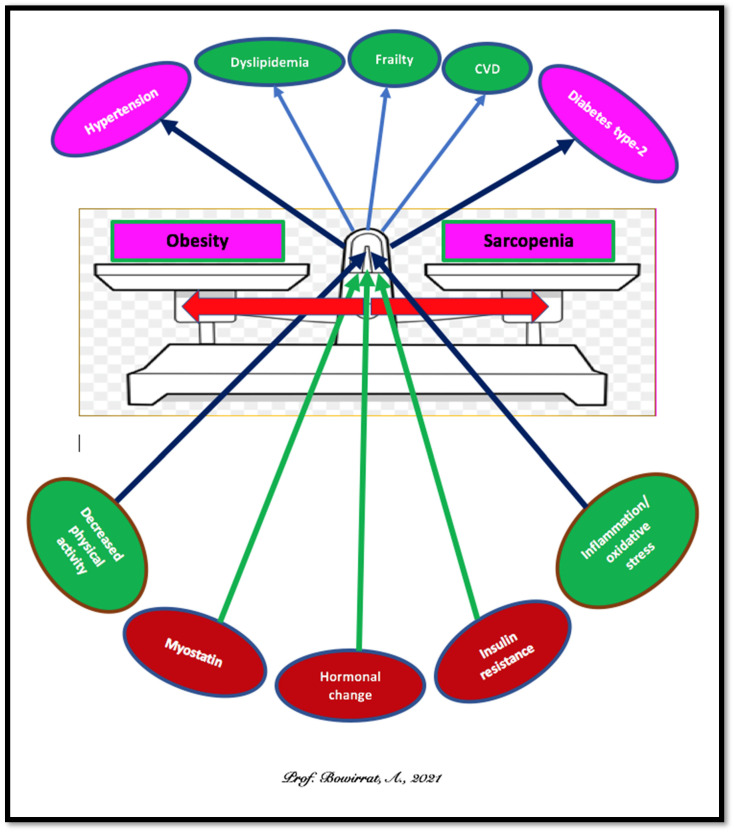
Mechanisms, consequences of Sarcopenia and obesity.

It is known that aging induces changes in body composition, such as an increase in visceral fat and reduced muscle mass. There is an emerging concept that sarcopenic obesity reflects the combination of Sarcopenia and obesity. Sarcopenia and obesity share several pathophysiological mechanisms, including the dangerous relationship between diminished muscle mass and strength in cardiometabolic disease mortality [73] (see Figure 4) [66].

**Figure 5:**
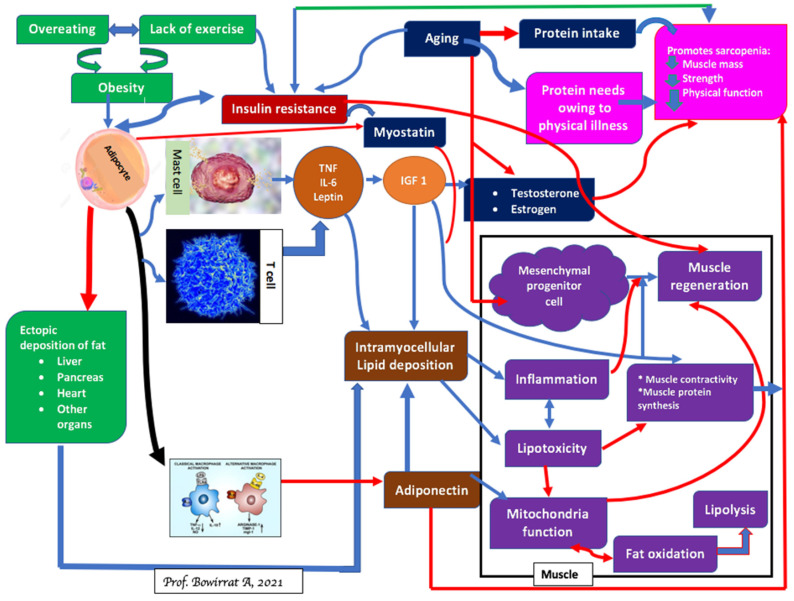
A proposed model of mechanisms leading to sarcopenic obesity.

The prevalence of obesity combined with Sarcopenia is rising in adults aged 65 years and older (see Figure 5 [67]) [74]. Typical treatment modalities include but are not limited to: (I) calorie restriction [75]; (II) protein supplementation [76]; (III) resistance and aerobic exercises [77,78,79]; (IV) testosterone, selective androgen receptor modulators [80]; (V) myostatin inhibitors [81]; (VI) ghrelin analogs [82]; (VII) vitamin K [83]; (VIII mesenchymal stem cell therapy [84] and precision behavioral management (IX).

## 6. Summary

Nutraceutical interventions hold great promise in treating genetically mediated physiological processes that affect metabolic functions and behaviors that contribute to metabolic dysfunction. The nutraceutical formula presented herein can reduce maladaptive carbohydrate craving while improving healthy fat loss, body composition, and overall wellness. This unique natural formulation improves weight management outcomes via integrating an individual’s unique heritable characteristics that mediate epigenetic expression and scientifically established nutritional principles. The synergy of a treatment coupling genetic and nutritional factors can [85,86,87] enhance fat metabolism and enhance muscle composition via improved hormone and neurotransmitter balance and the efficiency of nutrient function within the body.

In contrast to single health target approaches utilized by various pharmaceuticals, we propose using a balanced approach to health optimization by targeting multiple synergistic physiological systems. We believe that a balanced approach considering neurological, immunological, and metabolic pathways will enable clinicians to effectively modulate lipogenesis by increasing lipolysis while concurrently reducing maladaptive carbohydrate cravings.

Our approach will enhance wellness safely and predictably through the use of a Genetic Positioning System (GPS) Map [11]. In addition, Blum’s laboratory is in the process of developing an accurate Genetic Obesity Risk System (GORS) test consisting of many polymorphic risk alleles.

**Figure 6:**
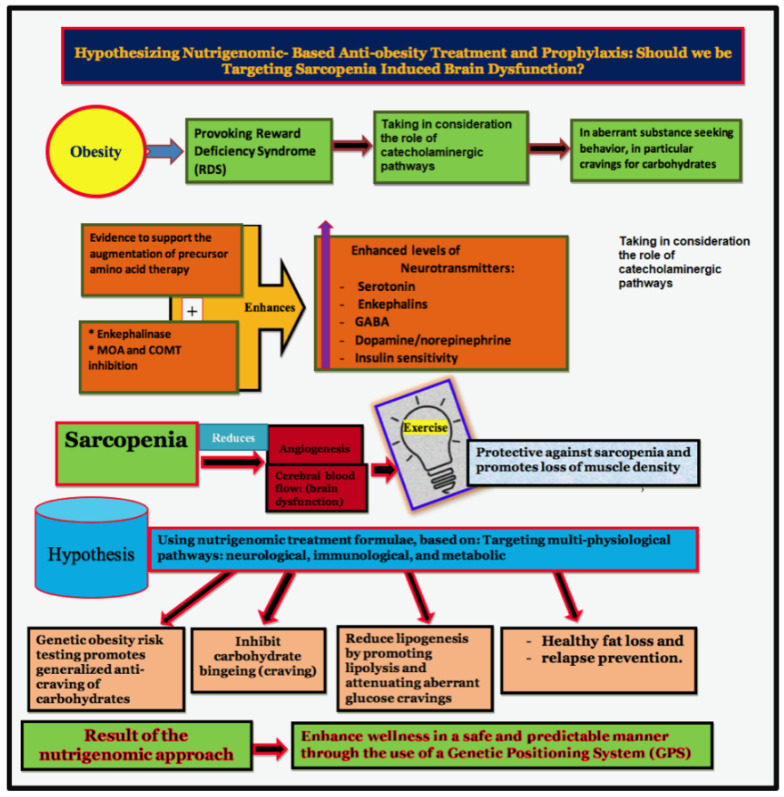
Hypothesizing nutrigenomic-based anti-obesity treatment and prophylaxis: should we be targeting sarcopenia-induced brain dysfunction?

While presently incomplete, the GPS Map will ultimately serve as a blueprint for personalized medicine in treating obesity and the development of strategies to reduce many harmful addictive behaviors and promote optimal health by using substances compatible with the body’s immune system. We encourage clinicians to contemplate using DEXA in place of BMI alone or calibrate with leptin levels, as mentioned in Shah and Braverman [35]. Combining exercise [88] with other previously listed modalities may help target Sarcopenia (see Figure 6, our model).

Interestingly Laparoscopic Sleeve Gastrectomy (LSG) induces long-term structural brain changes and modulates the sustained benefits of bariatric surgery in weight reduction. Associations between regional gray matter volume and resting-state functional connectivity (RSFC) suggest that LSG-induced structural changes contribute to RSFC change [89]. These results take on even more relevance in terms of potentially novel treatment options since our laboratory found evidence of enhancement of RSFC in both naïve rodents and heroin addicts [83,84]

Figure 6 summarizes our expert opinion with a precise basis for the potential early identification and treatment of obesity as a subset of RDS as a hypodopaminergic trait (genetic) or state (epigenetic). The authors are proposing a paradigm shift embracing nutrigenomic principles.

## 7. Conclusions

The genetic basis and future genetic testing of children for risk of aberrant generalized craving behavior are considered a prevention method. Here, we present evidence supporting the use of precursor amino acid therapy and modulation of enkephalinase, ***MOA***, and ***COMT*** inhibition in key brain regions. Such treatments manifest in improved levels of dopamine/norepinephrine, GABA, serotonin, and enkephalins. We also present evidence substantiating insulin sensitivity enhancement via Chromium salts, which affect dopamine neuronal synthesis regulation. We believe our unique combination of natural ingredients will influence many pathways leading to the promotion of well-being and normal healthy metabolic functioning. Sarcopenia has been shown to reduce angiogenesis and possible cerebral blood flow. Exercise seems to provide a significant benefit to overcome this obesity-promoting loss of muscle density.

Utilization of proposed nutrigenomic formulae based on coupling genetic obesity risk testing promotes generalized anti-craving of carbohydrates and can inhibit carbohydrate bingeing, inducing significant healthy fat loss and relapse prevention.

## Figures and Tables

**Table 1:** Systems to improve weight management.

The biochemical mechanisms involved in nutrition and energy management regulating intake, expenditure and storage controls and feedback;
2. Attenuation of the effects of chronic stress and inflammation (which overburden the endocrine system and can cause things such as excessive cortisol production) reducing fat storage;
3. The pleasure-seeking needs and reward circuitry of the brain, influencing psychological and emotional need-induced food cravings;
4. Promotion and support of healthy immune system function (involved in catalyzing survival response to metabolic threats; and
5. Supporting and maintaining optimal health of the neuroendocrine system through which the majority of metabolic signaling is processed.

## Data Availability

Not Applicable.
